# CombAlign: a code for generating a one-to-many sequence alignment from a set of pairwise structure-based sequence alignments

**DOI:** 10.1186/s13029-015-0039-1

**Published:** 2015-08-05

**Authors:** Carol L. Ecale Zhou

**Affiliations:** Computational Biology Group, Global Security Computing Applications Division, Lawrence Livermore National Laboratory, 7000 East Avenue, Livermore, CA 94550 USA

**Keywords:** Multiple sequence alignment, Multiple structure alignment, Matrix protein, Msa, Mssa, VP40, Secreted glycoprotein, sGP, Ebola

## Abstract

**Background:**

In order to better define regions of similarity among related protein structures, it is useful to identify the residue-residue correspondences among proteins. Few codes exist for constructing a one-to-many multiple sequence alignment derived from a set of structure or sequence alignments, and a need was evident for creating such a tool for combining pairwise structure alignments that would allow for insertion of gaps in the reference structure.

**Results:**

This report describes a new Python code, CombAlign, which takes as input a set of pairwise sequence alignments (which may be structure based) and generates a one-to-many, gapped, multiple structure- or sequence-based sequence alignment (MSSA). The use and utility of CombAlign was demonstrated by generating gapped MSSAs using sets of pairwise structure-based sequence alignments between structure models of the matrix protein (VP40) and pre-small/secreted glycoprotein (sGP) of Reston Ebolavirus and the corresponding proteins of several other filoviruses. The gapped MSSAs revealed structure-based residue-residue correspondences, which enabled identification of structurally similar versus differing regions in the Reston proteins compared to each of the other corresponding proteins.

**Conclusions:**

CombAlign is a new Python code that generates a one-to-many, gapped, multiple structure- or sequence-based sequence alignment (MSSA) given a set of pairwise sequence alignments (which may be structure based). CombAlign has utility in assisting the user in distinguishing structurally conserved versus divergent regions on a reference protein structure relative to other closely related proteins. CombAlign was developed in Python 2.6, and the source code is available for download from the GitHub code repository.

**Electronic supplementary material:**

The online version of this article (doi:10.1186/s13029-015-0039-1) contains supplementary material, which is available to authorized users.

## Background

In order to better define regions of similarity between a protein of interest and other closely related proteins, it is helpful to identify the residue-residue correspondences between each residue in the reference structure and the corresponding residues in each of the compared structures, given some pre-defined measure of correspondence. Residue-residue correspondences can be readily extracted from pairwise structure-based alignments, yielding correspondences in space, which may differ from those obtained by aligning proteins at the sequence level, or even differing from those obtained using standard multiple structure-based alignment programs [1, 2], as these may adjust local alignments between any two proteins in order to refine a consensus or define an optimal simultaneous alignment for the set.

Multiple sequence alignment (MSA) programs have a variety of uses, including classification of proteins, furthering the understanding of evolutionary conservation among proteins, detection of motifs, and prediction of function by homology [3, 4]. MSA algorithms comprise a variety of computational approaches and scoring functions, and generally involve identifying locally similar fragments (vertical equivalency) while merging pairwise alignments into a consensus (horizontal equivalency) [1, 5]. One could reasonably summarize the process as a means for generating an optimal hypothetical consensus structure against which each protein is readily aligned. However, there exist applications in which the end goal is not to drive a set of structures toward a consensus, but rather to contrast a single structure (or an aligned set of nearly identical structures) to a set of closely related, but notably different structures [6]. Few codes exist for constructing a one-to-many structure-based sequence alignment derived from a set of pairwise structure-based sequence alignments, and no open-source code was found that generated an alignment allowing for gaps to be inserted into the reference sequence. Thus, in this work, a code was created to perform this task. The code was then applied to help identify structure features that distinguish two proteins of Reston Ebolavirus (a species that is not pathogenic to human) from the corresponding proteins of several other closely related pathogenic filoviruses.

Because the Reston strain shares so much sequence identity (74 %-80 %) to its highly pathogenic sisters (i.e., Bundibugyo, Sudan, Tai Forest, Zaire), yet has not caused disease upon infection in humans, it presents a relevant case study for performing sequence and structure comparisons among its gene products. Although the present work was not scoped to conduct a comparative study between Reston and its near-neighbor species, selection of the viral matrix protein (VP40) and the pre-small/secreted glycoprotein (sGP; including the signal peptide) to demonstrate the utility of CombAlign was motivated by the important functions of these proteins in the life cycle of Filoviruses [7] and by interest in contrasting a non-pathogenic (to human) virus to a set of closely related pathogenic viruses.

The Zaire Ebolavirus VP40 protein is multifunctional, being involved in various processes in the virus’s life cycle, including virus particle assembly, budding from the host plasma membrane, interaction with the viral glycoprotein, binding to the ribonucleoprotein complex, and interaction with human cellular factors (see [8] and references therein). A functional feature of note includes the overlapping N-terminal PTAP/PPEY motifs [9], which facilitate the budding from the host cell membrane. The secreted glycoprotein is believed to function in host immune evasion, and its C-terminal delta peptide (a cleavage product), may prevent superinfection of infected cells [10–12]. Thus, the VP40 and sGP proteins were selected as test cases for demonstration of one-to-many comparisons using CombAlign.

## Results

### Development of CombAlign

A new code, CombAlign, was developed using Python 2.6. CombAlign takes as input a set of pairwise structure-based sequence alignments and generates a one-to-many, gapped, multiple structure-based sequence alignment (MSSA, see Methods) whereby the user can readily identify regions on the reference structure that have residue-residue correspondences with each of the other proteins against which the reference was structurally aligned. Although the intent in developing CombAlign was to construct multiple-sequence alignments from structure data, the code is agnostic to the program that is used to generate pairwise alignments used as input. However, because structure-based alignments can reveal structural (and, hence, potential functional) differences between proteins that may not necessarily be revealed through sequence-based alignments, development of CombAlign was targeted toward facilitating construction of multiple alignments using formats produced by two common protein structure tools: TM-align [13] and DaliLite [14].

CombAlign comprises a script (combAlign.py) that reads in the fasta sequence of a reference protein followed by a series of pairwise alignments, then creates an alignment object (alignment.py), which is used to combine the alignments into an MSSA, and lastly prints the results to a file. The reference fasta is used as a framework for recording correspondences between residues of the reference structure and residues of each structure in the comparison set; a data structure captures each position/residue in the reference fasta and tags it with a list of corresponding residues, one residue from each aligned structure (or ‘null’ if residue is absent or not aligned). Additionally, for each pairwise alignment, corresponding residues in the compared structure and positions of non-correspondence (gaps in the compared structure) are recorded; gaps that occur in the reference structure relative to the compared structure are inserted as null positions in a list attached to the preceding residue in the reference fasta sequence framework. Gap positions that occur in the reference structure relative to more than one compared structure are merged so as to avoid redundant gap insertion. The resulting one-to-many, gapped MSSA is formatted for output by dividing the reference fasta framework into segments corresponding to a user-provided or default line-size parameter and is printed to an output file. The correspondence data from the input pairwise alignments are reflected in the output MSSA. Symbols (‘-‘, ‘:’, ‘.’, ‘|’, “) used in CombAlign output have meaning identical to those of the program used to generate the pairwise alignments, and generally indicate the degree with which the residues corresponded. No other data provided by the pairwise alignment method (e.g., scoring, secondary structure prediction) are used by CombAlign.

### Test case 1: One-to-many alignment of virus matrix proteins (VP40s)

**Fig. 1:**
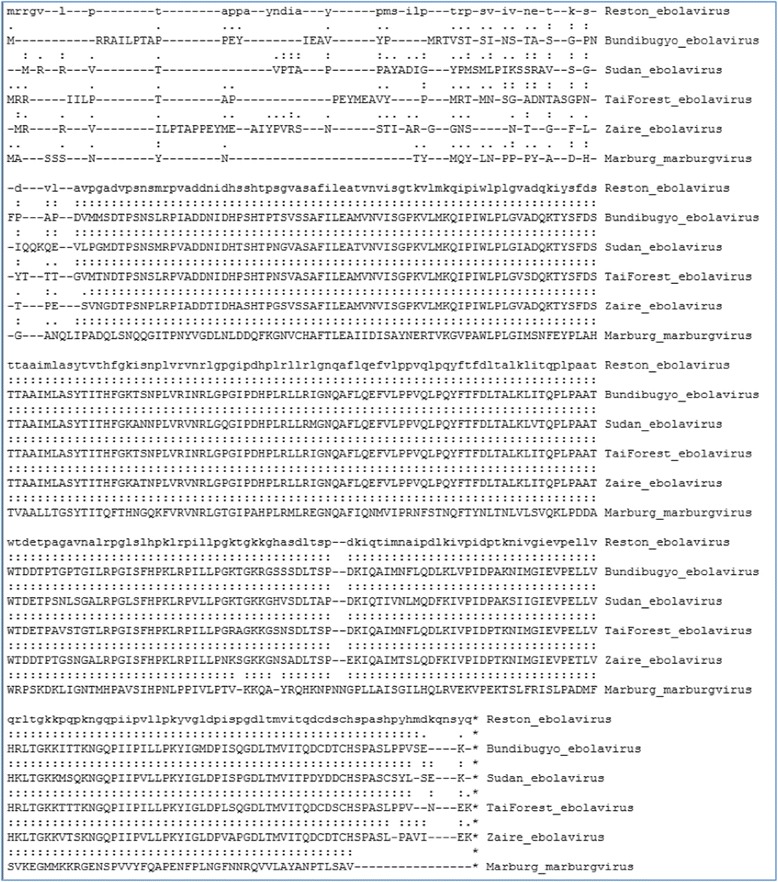
Multiple structure-based sequence alignment (MSSA) of Reston Ebolavirus VP40 model (reference) aligned with VP40 models from four Ebolaviruses and one Marburgvirus species. Pairwise TM-align alignments were combined using combAlign.py

The use and utility of CombAlign was demonstrated by generating a gapped MSSA using a structure model of the matrix protein (VP40) from Reston Ebolavirus (as the reference structure) and pairwise alignments between the reference and structure models of the VP40s from Bundibugyo, Sudan, Tai Forest, and Zaire Ebolaviruses and Marburg Marburgvirus (Fig. 1). The gapped MSSA revealed structure-based residue-residue correspondences between Reston Ebolavirus VP40 and each of the other VP40 proteins, which enabled identification of structurally similar versus differing regions in Reston compared to each of the closely related proteins.

In examing the MSSA (Fig. 1), it is apparent that the VP40 models are highly similar at the structure level, although clear differences emerge at the N- and C-termini, and small interruptions in correspondence are seen between the Reston Ebolavirus protein and that of Marburg Marburgvirus. The most apparent differences are observed within the N- and C- terminal regions. The mostly conserved PTAP/PPEY motifs (conserved in sequence among the Ebolaviruses but absent in the Marburgvirus protein), were disrupted in the pairwise structure alignments, and were, thereby, also distributed among gaps in the CombAlign MSSA. A distinguishing feature of the Reston Ebolavirus protein in comparison to each other protein was found to be the additional 5 residues at the extreme C-terminus (qnsyq), which are absent from all of the other VP40s. As this terminal region is believed to function in virus budding, the additional 5 residues in the Reston protein may have an adverse effect on VP40 function in this regard [9, 15, 16].

### Test case 2: One-to-many alignment of ebolavirus Pre-small/secreted glycoproteins (sGPs)

**Fig. 2:**
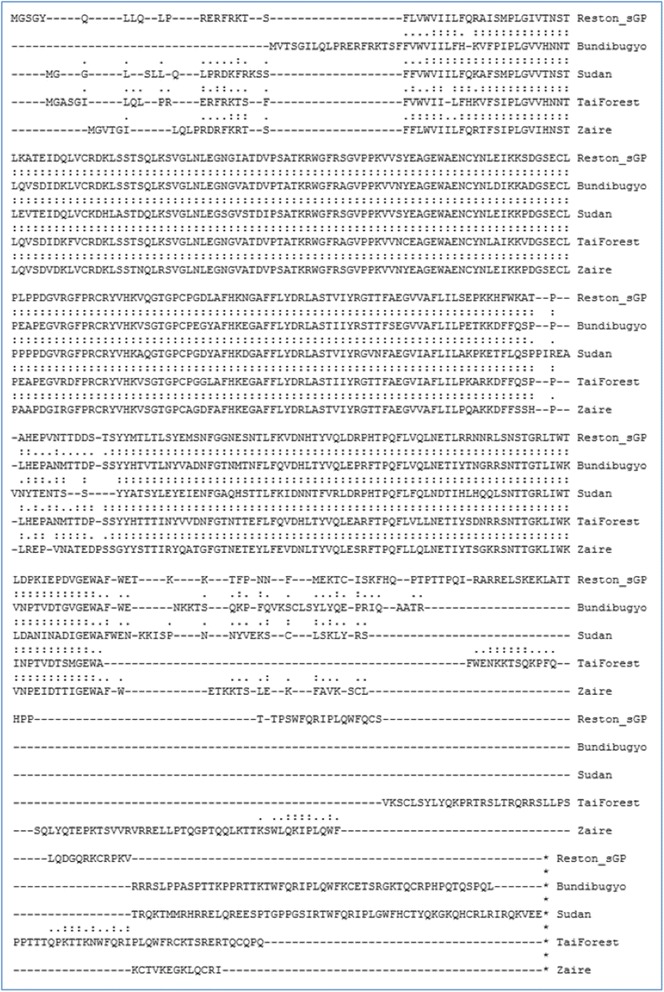
Multiple structure-based sequence alignment (MSSA) of Reston Ebolavirus secreted glycoprotein (sGP) model (reference) aligned with sGP models from four Ebolaviruses. Pairwise TM-align alignments were combined using combAlign.py

**Fig. 3:**
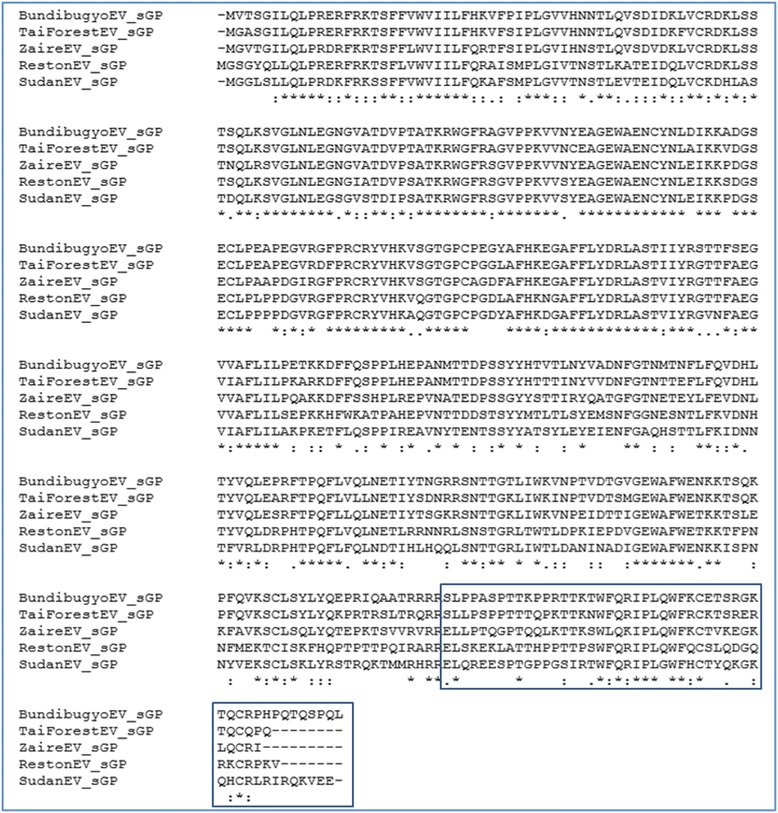
Ebolavirus secreted glycoproteins (sGPs) aligned with Clustal Omega. Box: delta peptide

A second test case involving structure-based comparison of Reston Ebolavirus sGP with the corresponding proteins from several other Ebolavirus species (Fig. 2) illustrates that combining structure-based alignments can reveal structural (and therefore potential functional) differences that might not be apparent using sequence-only methods (Fig. 3). The CombAlign alignment in Fig. 2 suggests that there may be considerable structural differences between sGP of Reston Ebolavirus compared to its pathogenic near neighbors in the N- terminal region, in the approximate center of the peptide chain, and in a large portion of the C-terminus, whereas the Clustal Omega [17] alignment depicted in Fig. 3 implies tight global and local correspondences between the residues of these proteins. Of particular note is the divergence seen at the C terminus, which contains the delta peptide (Fig. 3, box). This region is perfectly aligned at the sequence level, yet displays poor structural homology when examined using structure tools. Corresponding MSSAs were constructed using CombAlign to determine whether any given Ebolavirus sGP (as the reference structure) displayed close structure homology to any other (data not shown), and none was found to align well to any other. This apparent poor structure homology may be due to disorder in this region of the protein. Nonetheless, the MSSA in Fig. 2 supports the use of CombAlign for detecting structural deviations in a protein of interest relative to its structural near neighbors. It has been postulated that the delta peptide may function either to prevent superinfection of producer cells during early stages of infection or they may prevent trapping of budding progeny virus [11]. As the function of the delta peptide may be critical to pathogenicity or disease progression, it is interesting to note the apparent structural differences among the sGPs from the species depicted in Fig. 2, and based on this observation it would be reasonable to justify structure-function studies of these peptides in the context of their proposed functions.

### Code availability and requirements

The CombAlign source code is available for download from the GitHub code archive. To access the code, one should first download and install the git client [18, 19]. The CombAlign project files can be cloned either using the GUI interface or more simply from the command line (once the software is installed, typing ‘git’ should display a help menu). CombAlign files can then be downloaded by entering, “git clone https://github.com/carolzhou/Protein”. CombAlign was written in Python 2.6 and can be run on any desktop or server that supports Python. No specific processing requirements are indicated. A help menu is provided by typing, “python combAlign.py help”.

## Discussion

CombAlign is a new Python code that generates a gapped, multiple structure-based sequence alignment (MSSA) given a set of pairwise structure-based sequence alignments. CombAlign has utility in assisting the user in distinguishing structurally conserved versus divergent regions on a reference protein structure relative to other closely related structures. The method for combining multiple pairwise alignments is straightforward, involving the recording of pre-computed residue-residue correspondences between positions on the reference protein and each compared structure, and insertion of non-redundant gaps, as needed, to reflect amino-acid deletions or structural divergence in the reference relative to one or more compared structures.

CombAlign is not intended for use in applications for which greater benefit would be provided using a multiple structure alignment as generated by the vast majority of open-source programs [20], nor does it propose to address matters of protein evolution or function prediction through homology. Rather, CombAlign finds application in questions involving detection of structure features or motifs that may distinguish a protein of interest from a set of related proteins [6]. CombAlign may also find application in bioinformatics pipelines that run in high throughput, making use of parallel processing to simultaneously perform pairwise alignments, which can then be rapidly combined. Furthermore, when using software for multiple sequence alignments, users may at times submit subsequent jobs that differ only slightly. If only a few sequences are replaced compared to a previous job, most computational results could be "recycled" using CombAlign, whereas this approach would not work using consensus structure-based algorithms.

The test cases presented here demonstrate such an application in contrasting Reston Ebolavirus VP40 and sGP with corresponding proteins from several closely related pathogenic species. Not surprisingly, the most apparent differences in VP40 were observed at the N- and C- terminal regions (Fig. 1), which suggests regions of relatively disordered structure. However, the absence of 5 residues at the C-terminal region of the reference protein relative to the proteins in the comparison set were apparent from the MSSA and allowed one to verify that these residues were indeed a distinguishing feature of the reference protein (Test Case 1). The pronounced structural deviation in the C-terminal portion of the sGP protein of Reston Ebolavirus relative to the sGPs of the pathogenic species (Test Case 2) was evident from the CombAlign output (Fig. 2), whereas these potential differences were not apparent from a sequence-based multiple alignment (Fig. 3), supporting the utility of CombAlign in the detection of putative distinguishing features that may help to explain differential phenotype. Furthermore, by using the reference protein’s fasta sequence as a framework upon which to build the MSSA, it is a convenience that residues at the N- and C-terminal ends of the reference protein are always included in the final alignment, regardless of whether they have failed to structurally align within the pre-defined cutoffs of the pairwise alignment program, or whether those residues failed to be assigned coordinates during crystallization or structure modeling.

Although there exist crystal structures for several of the filovirus VP40 proteins [21] (Sudan: 3TCQ, 4LD8; Zaire: 4LDB, 4LDD, 4LDM; Ebola sp.: 1ES6, 1H2C, 1H2D), structure models for all proteins used in this demonstration were generated using I-TASSER [22]. Hypothetical models for proteins that are represented in the Protein Data Bank database (PDB) will naturally be generated using those entries as primary templates, but may in some cases be more complete due to fragment assembly relying on alternate templates or loop building methods [4, 22]. Furthermore, although models for similar protein sequences not represented in the PDB will usually be modeled using primarily the same templates, they will not necessarily yield identical structure models with respect to side-chain or backbone positioning [4, 22]. For this reason, and for consistency, all of the VP40 and sGP sequences in this demonstration were modeled using I-TASSER. Also, TMalign [13] and DaliLite [14] were used for structure alignments used in the test cases, although any sequence or structure alignment code could be used to generate the input data set, provided that either the TMalign or DaliLite output format is followed.

## Conclusions

CombAlign fills a modest niche in the field of protein structure and sequence alignment by providing a means for comparing, on a one-to-many basis, a single protein structure with a set of closely related structures. CombAlign was developed in Python 2.6 on a Linux cluster running RedHat release 6.5 and is available for download from github.

## Methods

### Structure modeling and alignment

The following sequences were downloaded from Genbank and used in this work: 1) Matrix protein (VP40) sequences for Ebolavirus species Reston (NP_690582.1), Bundibugyo (YP_003815434.1), Sudan (YP_138522.1), Tai Forest (YP_003815425.1), and Zaire (NP_066245.1), and Marburgvirus species Marburg (YP_001531155.1); 2) secreted glycoprotein (sGP) sequences for Ebolavirus Reston (NC_004161.1), Bundibugyo (NC_014373.1), Sudan (NC_006432.1), Tai Forest (NC_014372.1), and Zaire (NC_002549.1); 3) glycoprotein (GP) sequences for Ebolavirus Reston (NP_690583.1), Bundibugyo (NC_014373.1), Sudan (NC_006432.1), Tai Forest (NC_014372.1), and Zaire (NC_002549.1). Protein structure models were generated for each VP40, sGP, and GP sequence using the I-TASSER on-line [22, 23]. Results sets were downloaded and unpacked, and, for each modeled sequence, model1 was selected for further analysis. Data are shown for VP40 and sGP (Figs. 1, 2, 3); data for GP are provided as supplementary files only.

Pairwise alignments between the VP40 and sGP models from Reston Ebolavirus (as the reference structures) and the corresponding models from each of the other species were generated using the TM-align on-line server [24] or the DaliLite on-line server [25]. Each alignment was copied from the web-page results or downloaded to a local directory and saved to a text file. The multiple sequence alignment was generated using the Clustal Omega on-line server [26]. The input files to CombAlign consisted of two files (one for each of VP40 and sGP data) to which were prepended the fasta sequence of Reston Ebolavirus Vp40 or sGP, respectively (see supplementary data, “Additional file 1” and “Additional file 2”). The outputs from running CombAlign using these input files comprise “Additional file 3” and “Additional file 4” and are summarized in Figs. 1 and 2, respectively. An additional MSSA of Reston Ebolavirus glycoprotein (GP) is provided using pairwise alignments generated using DaliLite (see supplementary data, “Additional file 5” and “Additional file 6”) to provide the user with a sample input file in DaliLite format and to demonstrate that combAlign.py correctly processes that format. “Additional file 7” (in Supplementary Data) provides an example of CombAlign output in alignedFASTA format.

## Additional files

Additional file 1:
**Sample input file 1 to CombAlign.** Fasta sequence of Reston Ebolavirus VP40 protein followed by pairwise alignments of Reston Ebolavirus VP40 with VP40s from several other filoviruses and one Marburgvirus, in TM-align format. (TXT 5 kb) Additional file 2:
**Sample input file 2 to CombAlign.** Fasta sequence of Reston Ebolavirus sGP protein followed by pairwise alignments of Reston Ebolavirus sGP with sGPs from several other filoviruses, in TM-align format. (TXT 5 kb) Additional file 3:
**Sample output file 1 from CombAlign.** One-to-many, gapped, multiple structure-based sequence alignment of Reston Ebolavirus VP40 compared with VP40s of several filoviruses and one Marburgvirus, in combAlign format. (ZIP 1 kb) Additional file 4:
**Sample output file 2 from CombAlign.** One-to-many, gapped, multiple structure-based sequence alignment of Reston Ebolavirus sGP compared with sGPs of several Ebolaviruses, in combAlign format. (ZIP 1 kb) Additional file 5:
**Sample input file 3 to CombAlign.** Fasta sequence of Reston Ebolavirus GP protein followed by pairwise alignments of Reston Ebolavirus GP with GPs from several other Ebolaviruses, in DaliLite format. (TXT 17 kb) Additional file 6:
**Sample output file 3 from CombAlign.** One-to-many, gapped, multiple structure-based sequence alignment of Reston Ebolavirus GP compared with GPs of several Ebolaviruses, in combAlign format. (ZIP 2 kb) Additional file 7:
**Sample output file 4 from CombAlign.** One-to-many, gapped, multiple structure-based sequence alignment of Reston Ebolavirus GP compared with GPs of several Ebolaviruses, in alignedFASTA format. (ZIP 2 kb)
